# Differential expression and co-expression gene networks reveal candidate biomarkers of boar taint in non-castrated pigs

**DOI:** 10.1038/s41598-017-11928-0

**Published:** 2017-09-22

**Authors:** Markus Drag, Ruta Skinkyté-Juskiené, Duy N. Do, Lisette J. A. Kogelman, Haja N. Kadarmideen

**Affiliations:** 1 Department of Veterinary and Animal Sciences, Faculty of Health and Medical Sciences, University of Copenhagen, Grønnegårdsvej 7, Frederiksberg C, 1870 Denmark; 2 Department of Animal Science, McGill University, 21111 Lakeshore Road, Ste-Anne-de-Bellevue, H9X 3V9 QC Canada; 3 Danish Headache Center, Department of Neurology, Rigshospitalet Glostrup, Nordre Ringvej 67, Glostrup, 2600 Denmark; 4 Section of Systems Genomics, Department of Bio and Health Informatics, Technical University of Denmark, Kemitorvet, Building 208, 2800 Kgs. Lyngby, Denmark

## Abstract

Boar taint (BT) is an offensive odour or taste observed in pork from a proportion of non-castrated male pigs. Surgical castration is effective in avoiding BT, but animal welfare issues have created an incentive for alternatives such as genomic selection. In order to find candidate biomarkers, gene expression profiles were analysed from tissues of non-castrated pigs grouped by their genetic merit of BT. Differential expression analysis revealed substantial changes with log-transformed fold changes of liver and testis from −3.39 to 2.96 and −7.51 to 3.53, respectively. Co-expression network analysis revealed one module with a correlation of −0.27 in liver and three modules with correlations of 0.31, −0.44 and −0.49 in testis. Differential expression and co-expression analysis revealed candidate biomarkers with varying biological functions: phase I (*COQ3*, *COX6C*, *CYP2J2*, *CYP2B6*, *ACOX2*) and phase II metabolism (*GSTO1*, *GSR*, *FMO3*) of skatole and androstenone in liver to steroidgenesis (*HSD17B7*, *HSD17B8*, *CYP27A1*), regulation of steroidgenesis (*STARD10*, *CYB5R3*) and GnRH signalling (*MAPK3*, *MAP2K2*, *MAP3K2*) in testis. Overrepresented pathways included “Ribosome”, “Protein export” and “Oxidative phosphorylation” in liver and “Steroid hormone biosynthesis” and “Gap junction” in testis. Future work should evaluate the biomarkers in large populations to ensure their usefulness in genomic selection programs.

## Introduction

Boar taint (BT) is an offensive odour that occurs in cooked or heated pork from a proportion of non-castrated male pigs. Two compounds are mainly responsible for BT: skatole (3-methyl-indole) and androstenone (5α-androst-16-ene-3-one)^1,2^. Skatole is a by-product of enzymatic breakdown of L-tryptophan by bacterial action in the large intestine and subsequently absorbed into the blood^3^. Androstenone is a steroid produced in the testis where it is transported by blood to the salivary gland and functions as a mating pheromone^4^. It is synthesised from pregnenolone in association with sexual maturity together with other testicular steroids, such as testosterone and oestrogens^5,6^. Due to their lipophilic properties, the compounds accumulate in the adipose tissue and concentrations are correlated with the level of sexual maturity at the time of slaughter^7,8^ and/or the intensity of liver degradation^9,10^. A distinct relationship between androstenone and skatole exists: high concentration of androstenone prevents breakdown of skatole by inhibiting enzymes responsible for skatole metabolism and causes accumulation in adipose tissues^11^. Furthermore, androstenone itself is an active contributor to BT, and it has been proposed that elevated levels of androstenone are primary to elevated levels of skatole in the hierarchy of BT development^12^. Threshold levels for skatole and androstenone concentrations in fat considered to cause BT are 0.5 to 1 µg/ g for androstenone and 0.20 to 0.25 µg/g for skatole^2^.

Boar taint is the main factor preventing the production of non-castrated male pigs in many countries^13^ and often leads to depreciation of the carcass, even though their production is associated with a number of significant economic advantages including increased efficiency, leaner carcasses and lower faecal and urinary nitrogen losses^14^. Furthermore, production of non-castrated males is often viewed as advantageous in the context of animal welfare due to the absence of surgical castration. Indeed, this practice is often performed without anaesthesia or analgesia which results in pain and stress for the animal^15^, but the practice is justified by its alleviation of aggression and sexual activity which can result in leg fractures and skin damage^16^. Surgical castration is also an effective technique for avoiding BT, but increased attention to the animal welfare issues has led to a voluntary initiative to end the practice in the European Community from January 1, 2018^17^.

In order to avoid BT in the production of non-castrated males without surgical castration, two overall solutions have been proposed: (i) chemicals and/or chemotherapeutics such as immunocastration and (ii) optimised animal breeding programs. For the first solution, immunocastration by Improvac^®^ (Pfizer Ltd, NY, USA) has been found to be an effective strategy against BT with no detrimental effects on carcass and meat quality^18–23^ and with environmental benefits such as increased or at least similar growth rates and feed conversion efficiencies between immunised and surgically castrated pigs^24^. However, the European Food Safety Authority (EFSA) has anticipated poor public acceptance of immunocastration^25,26^ due to the possible perception of a link with hormone treatment and possible harmful residues^24,27^.

For the latter solution, genomic-based breeding programs have been proposed. As skatole and androstenone are moderate to highly heritable traits^9,11,28–30^, reduction of BT would be possible through breeding programs. By this strategy, breeding organisations would apply genomic selection on individuals with desirable genotypes that are predisposed to low genetic merit of BT compounds without the need for trait recording^31^. Previous work has indeed established low and mostly favourable genetic correlations between BT compounds and production traits such as the quality of meat without negatively affecting important male fertility trait^32^. Thus, selection of low genetic merit of BT can be deemed an effective and sustainable solution. By employing molecular genetic methods, identification of male pigs with low genetic merit of BT is possible, before they actually develop the condition or have their traits recorded^33^. However, this requires knowledge on highly predictive key genes associated with the trait of interest. In turn, these genes can be applied as biomarkers, e.g. Mazzoni, *et al*.^34^ and Salleh, *et al*.^35^. Finding biomarkers with strong predictive powers for a trait can be greatly enhanced with omics technologies, such as transcriptomics^36^. By measuring changes in gene expression profiles, analysis of differentially expressed (DE) genes associated with BT has been performed in a variety of breeds and experimental designs (as reviewed by Zadinová, *et al*.^37^). While many studies employed microarrays to obtain gene expression profiles, a few employed RNA sequencing (RNA-Seq) which provides far more precise measurements of transcript expression levels and their isoforms than traditional technologies^38^. Another powerful analysis within transcriptomics is construction of co-expression networks which reveal co-expressed (CE) genes with a high connectivity (“hub genes”). Hub genes are often regulatory genes, with major impact on genetic networks and may affect the trait of interest^39–41^. To the authors knowledge, no studies have applied a systems biology approach^36^ to genetic merit of BT. By this approach, findings from DE and CE analyses from RNA-Seq data are integrated in order to enhance discriminatory power of highly associated genes, useful as candidate biomarkers within animal breeding.

**Figure 1:**
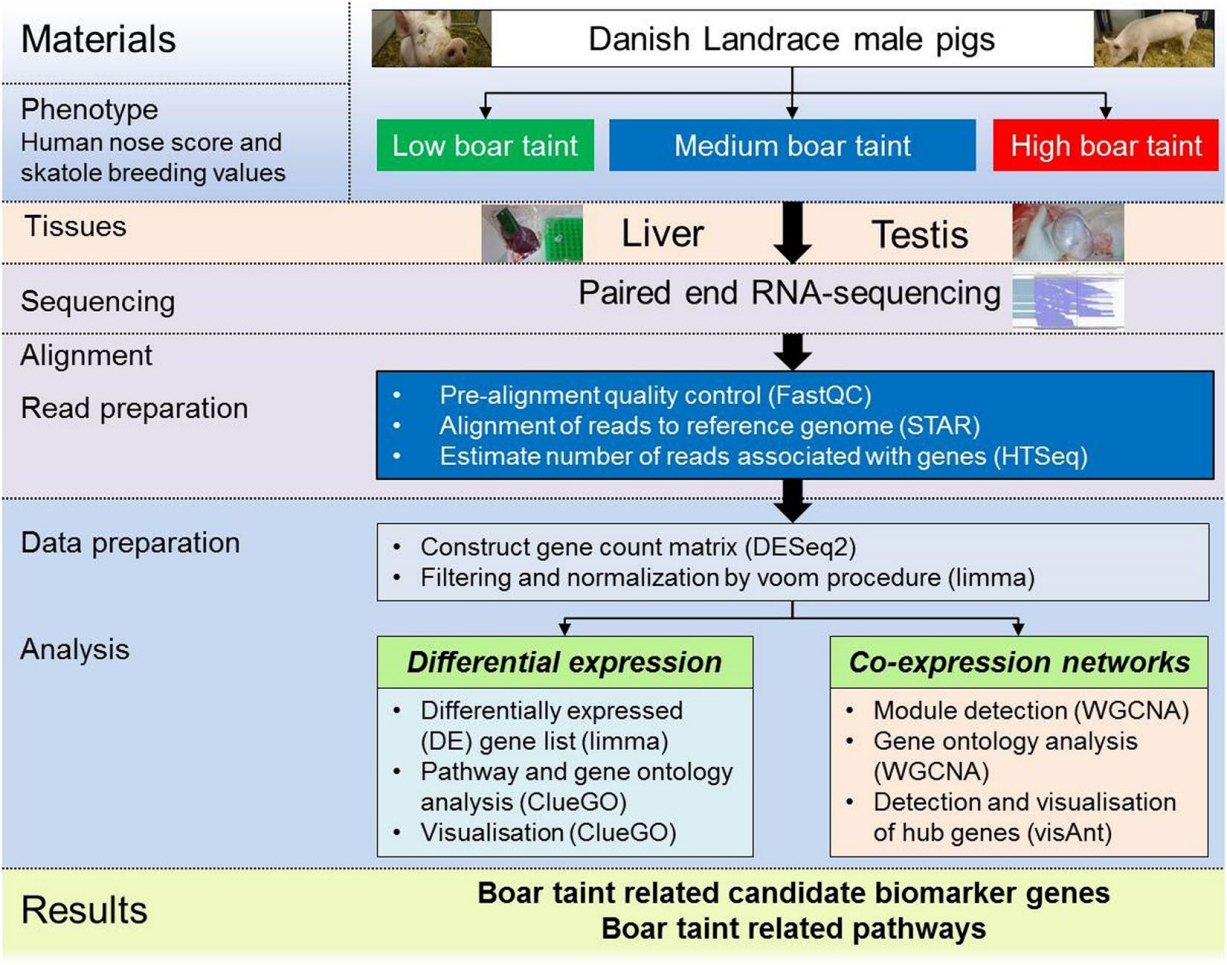
Illustration of the study design. From a total of 114 Landrace male pigs, 48 pigs were grouped as having low, medium and high boar taint (BT) according to estimated breeding values (EBVs) of skatole and human nose score of their respective sires. Each group consisted of 16 pigs. At 100 kg weight, they were slaughtered and two tissues were sampled. Of these, liver and testis tissues were subjected to paired-end RNA sequencing. These were analysed for differentially expressed (DE) genes and co-expressed (CE) genes which were extracted from co-expression networks by a range of statistical methods. In turn, this led to the discovery of candidate biomarkers for BT and BT related pathways.

The aims of this study were: (i) to detect important DE genes in gene expression profiles of liver and testis in male pigs associated with divergent genetic merit of BT, (ii) to build co-expression networks by application of the Weighted Gene Co-expression Network Analysis (WGCNA) method^42^ and extract important hub genes, (iii) to integrate results and extract candidate biomarkers associated with genetic merit of BT and evaluate their potential within animal breeding (Fig. 1).

## Results

### Genetic merit of boar taint

The animal model in this study comprised of commercial Danish Landrace male pigs (*n* = 114). The pigs were produced from sires with known genetic merit of BT assessed from estimated breeding values (EBVs) of skatole concentrations in fat and human nose scores (HNS). The genetic merit of BT was defined as the summarised EBVs calculated as the sum of EBVs of skatole and HNS obtained from the respective sire of each pig. In brief, EBVs are a measure of the relative genetic performance compared with a contemporary group of a trait and used as an industry standard in animal breeding. The full concept, definition and methods used to calculate EBVs are given in Bourdon and Bourbon^43^. Based on the distribution of summarised EBVs, a total of 48 pigs (16 low, 16 medium and 16 high) were selected and used in the analysis, representing a low, medium and high genetic merit of BT. The means of the summarised EBVs (±standard deviation) for each group were 0.71 (±0.19), −0.01 (±0.09) and −0.38 (±0.17) for high, medium and low genetic merit of BT, respectively. A list of selected animals and their EBVs are available in Supplementary file 1.

### RNA sequencing of liver and testis tissue

RNA-Seq was performed on liver and testis tissue of the selected pigs. Post-mapping quality control revealed that a mean of 33.25 million reads throughout the 96 samples had been successfully mapped to the reference genome. The number of reads (±SD) aligned to genes in liver and testis tissue were 7.76 (±1.61) million and 7.04 million (±2.44), respectively. Quality control revealed a distribution of 50.14% exonic reads, 41.29% intergenic reads and 8.57% intronic reads in liver and 47.42% exonic reads, 37.89% intergenic reads and 14.79% intronic reads in testis. After filtering of genes with expression levels equal to or fewer than five counts, the number of genes included in the differential expression and co-expression analyses were 10,545 for liver and 12,731 for testis, respectively.

### Identification of differentially expressed genes

Differential expression analysis of liver and testis by contrast of high *vs* low genetic merit of BT from the three-group study design revealed 507 and 5,943 differentially expressed (DE) genes (FDR < 0.05), respectively. An additional filtering step which compared expression profiles for each DE gene throughout the three groups revealed that 241 and 2,315 genes were significantly (*P* < 0.05) different across groups in liver and testis, respectively. The log-transformed fold change of the DE genes in liver and testis ranged from −3.39 to 2.96 and −7.51 to 3.53, showing substantial gene expression differences. The top significant DE genes were *MORN2*, *AASDHPPT*, *SPCS3*, *SEC*. *11* *C* and *NDUFC1* in liver and *LAMP1*, *TNPO1*, *TOPORS*, *GFPT1* and *COX4I1* in testis. The top 25 most significant DE genes found in liver and testis are presented in Table 1 and Table 2, respectively. Full result tables are included in Supplementary file 2.

**Table 1:** Top 25 differentially expressed (DE) genes from liver.

Gene	Description	logFC^a^	FDR^b^	K-W *P*.
*MORN2*	MORN repeat containing 2	−0.83	0.007	0.0043
*AASDHPPT*	aminoadipate-semialdehyde dehydrogenase-phosphopantetheinyl transferase	−0.62	0.009	0.011
*SPCS3*	signal peptidase complex subunit 3	−0.64	0.009	0.073
*SEC*. *11 C*	SEC11 homolog C, signal peptidase complex subunit	−0.73	0.009	0.024
*NDUFC1*	NADH:ubiquinone oxidoreductase subunit C1	−0.76	0.009	0.0079
*ZNF181*	zinc finger protein 181	−0.58	0.009	0.0012
*ARL5A*	ADP ribosylation factor like GTPase 5A	−0.66	0.009	0.0023
*NEURL1*	neuralized E3 ubiquitin protein ligase 1	−1.30	0.009	0.0053
*EIF1AY*	eukaryotic translation initiation factor 1A, Y-linked	−0.60	0.009	0.011
*SGPP1*	sphingosine-1-phosphate phosphatase 1	−0.74	0.009	0.0044
*PLGRKT*	plasminogen receptor, C-terminal lysine transmembrane protein	−0.65	0.009	0.024
*SFR1*	SWI5-dependent homologous recombination repair protein 1	−1.28	0.009	0.0049
*ATP5H*	ATP synthase, H+ transporting, mitochondrial Fo complex subunit D	−0.86	0.009	0.019
*KBTBD3*	kelch repeat and BTB (POZ) domain containing 3	−0.69	0.009	0.0068
*DUS4L*	dihydrouridine synthase 4-like	−0.59	0.009	0.02
*MED21*	mediator complex subunit 21	−0.62	0.009	0.015
*COMMD8*	COMM domain containing 8	−0.74	0.010	0.016
*SNX14*	sorting nexin 14	−0.61	0.010	0.058
*ACTR6*	ARP6 actin-related protein 6 homolog (yeast)	−0.60	0.010	0.056
*MOCS2*	molybdenum cofactor synthesis 2	−0.79	0.010	0.039
*PDCD10*	programmed cell death 10	−0.62	0.010	0.018
*ZBTB8OS*	zinc finger and BTB domain containing 8 opposite strand	−0.58	0.011	0.068
*METTL23*	methyltransferase like 23	−0.62	0.011	0.014
*NIPSNAP3B*	nipsnap homolog 3B (C. elegans)	−0.49	0.011	0.014
*IKBIP*	IKBKB interacting protein	−1.03	0.011	0.011

^a^LogFC = the log2 transformed fold change obtained from the limma procedure.

^b^FDR = the Benjamini-Hochberg (FDR) procedure adjusted *P* value obtained from the limma procedure.

^c^K-W *P*. = the *P* value obtained from Kruskal Wallis test of expression profile from each gene throughout the three groups.

Abbreviations: *LogFC* log transformed fold change, *FDR* False Discovery Rate, *K-W* Kruskal Wallis.

**Table 2:** Top 25 differentially expressed (DE) genes from testis.

Gene	Description	logFC^a^	FDR^b^	K-W *P*.
*LAMP1*	lysosomal-associated membrane protein 1	0.73	8.077E-06	0.015
*TNPO1*	transportin 1	−0.81	8.077E-06	1.20E-05
*TOPORS*	topoisomerase I binding, arginine/serine-rich, E3 ubiquitin protein ligase	−0.56	8.077E-06	7.10E-06
*GFPT1*	glutamine–fructose-6-phosphate transaminase 1	−0.82	1.139E-05	0.00019
*COX4I1*	cytochrome c oxidase subunit IV isoform 1	0.77	1.139E-05	0.059
*RLF*	rearranged L-myc fusion	−0.70	1.139E-05	7.70E-05
*C17orf62*	chromosome 17 open reading frame 62	0.91	1.139E-05	1.20E-05
*KIAA1109*	KIAA1109	−1.24	1.139E-05	0.00063
*RAB5C*	RAB5C, member RAS oncogene family	0.67	1.139E-05	0.008
*IRGC*	immunity-related GTPase family, cinema	0.99	1.139E-05	0.023
*LRPAP1*	LDL receptor related protein associated protein 1	0.52	1.181E-05	0.015
*MZT2A*	mitotic spindle organizing protein 2 A	0.93	1.181E-05	0.0054
*ZNF3*	zinc finger protein 3	−1.59	1.181E-05	0.00043
*KANSL1L*	KAT8 regulatory NSL complex subunit 1 like	−1.22	1.182E-05	0.00056
*EXOC5*	exocyst complex component 5	−1.05	1.302E-05	0.00043
*MAF1*	MAF1 homolog, negative regulator of RNA polymerase III	0.57	1.302E-05	3.00E-04
*PARPBP*	PARP1 binding protein	−1.54	1.302E-05	0.00051
*HLTF*	helicase-like transcription factor	−1.04	1.372E-05	0.00021
*ATR*	ATR serine/threonine kinase	−0.62	1.372E-05	5.50E-05
*ZYG11B*	zyg-11 family member B, cell cycle regulator	−1.19	1.372E-05	0.00077
*HNRNPAB*	heterogeneous nuclear ribonucleoprotein A/B	0.64	1.372E-05	0.015
*PFN1*	profilin 1	0.62	1.372E-05	0.0052
*EDF1*	endothelial differentiation-related factor 1	1.45	1.372E-05	0.029
*SLC25A39*	solute carrier family 25 member 39	0.86	1.372E-05	0.056
*CREBRF*	CREB3 regulatory factor	−1.41	1.372E-05	0.003

^a^LogFC = the log2 transformed fold change obtained from the limma procedure.

^b^FDR = the Benjamini-Hochberg (FDR) procedure adjusted *P* value obtained from the limma procedure.

^c^K-W *P*. = the *P* value obtained from Kruskal Wallis test of expression profile from each gene throughout the three groups.

Abbreviations: *LogFC* log transformed fold change, *FDR* False Discovery Rate, *K-W* Kruskal Wallis.

### Gene functional enrichment analysis

**Figure 2:**
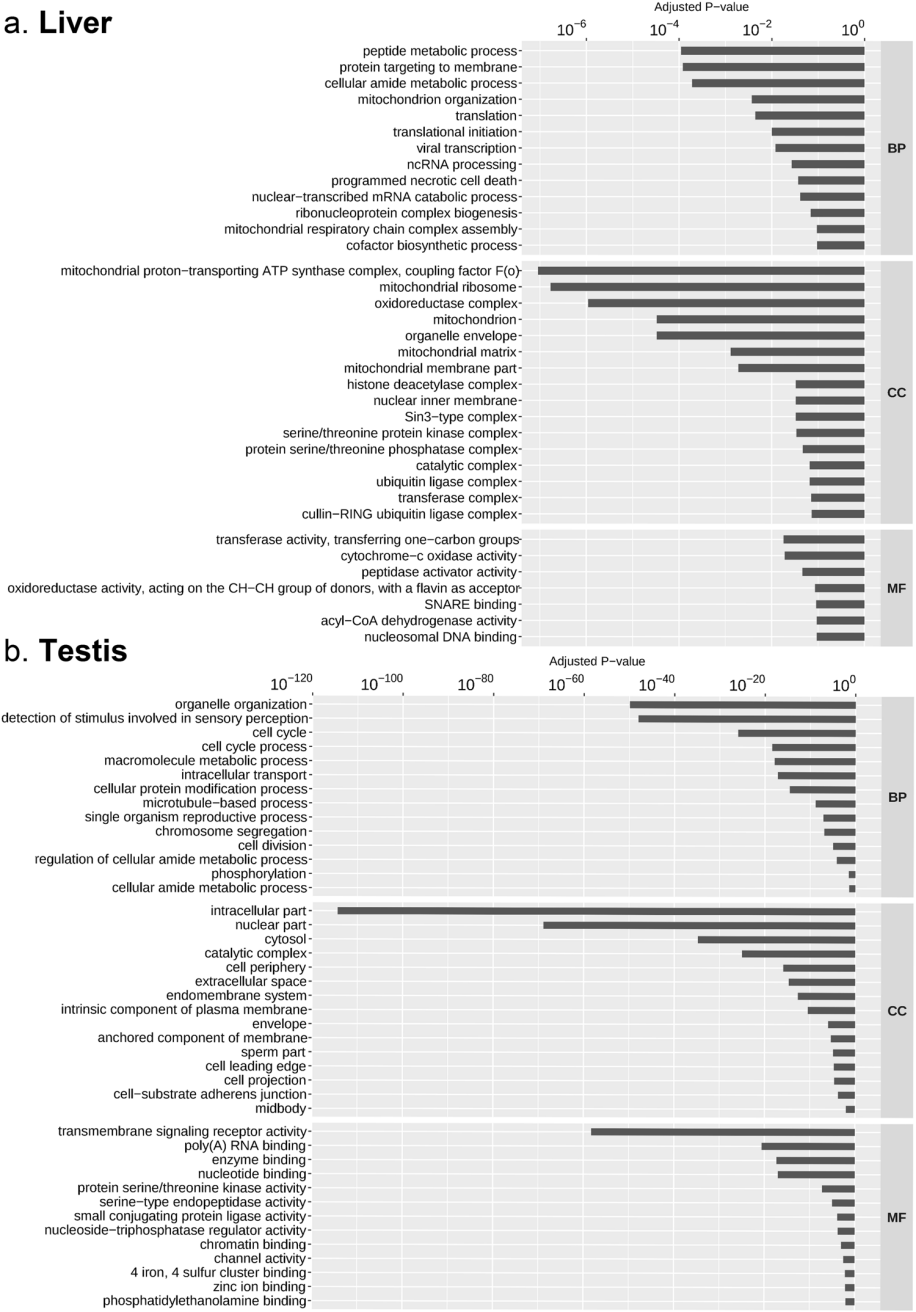
Bar plot of enriched gene ontology (GO) terms of (**a**) liver and (**b**) testis. Y-axis indicates the GO term, X-axis indicates the adjusted *P*-value. Abbreviations: *BP* Biological process, *CC* cellular component, *MF* molecular function.

Gene functional enrichment analysis revealed a total of 39 and 421 significantly enriched (Adj. *P*-val. < 0.05) gene ontology (GO) terms in liver and testis, respectively (Supplementary file 3). Subsequent filtering of redundant GO terms by semantic similarity further reduced the amount in order to visually represent the most enriched terms in each tissue (Fig. 2). In liver, “Peptide metabolic processes” (Adj. *P*-val. = 1.1 × 10^−4^) were the most significantly enriched GO terms of Biological Process (BP), “Mitochondrial proton-transporting ATP synthase complex, coupling factor F(o)” (Adj. *P*-val. = 0.9 × 10^−7^) were the most significantly enriched Cellular Component (CC) terms and”Transferase activity, transferring one-carbon groups” (Adj. *P*-val. = 1.8 × 10^−2^) were the most significantly enriched Molecular Function (MF) terms (Fig. 2a). In testis, the GO terms “Organelle organization” (Adj. *P*-val. = 1.8 × 10^−50^) were the most significantly enriched BP, “Intracellular part” (Adj. *P*-val. = 4.4 × 10^−115^) were the most significantly enriched CC and finally, “Transmembrane signalling receptor activity” (Adj. *P*-val. = 1.0 × 10^−61^) were the most significantly enriched MF (Fig. 2b). A total of 11 GO terms were found to be significantly (Adj. *P*-val. < 0.05) enriched in both tissues which comprised 5 BP and 6 CC terms. According to results obtained from each individual tissue, the most significantly enriched common GO terms were “Mitochondrion” (Adj. *P*-val. = 3.2 × 10^−5^), “Peptide metabolic process” (Adj. *P*-val. = 1.1 × 10^−4^) and “Cellular amide metabolic process” (Adj. *P*-val. = 1.9 × 10^−4^) in liver and “Catalytic complex” (Adj. *P*-val. = 1.0 × 10^−25^), “Transferase complex” (Adj. *P*-val. = 5.0 × 10^−20^) and “Ribonucleoprotein complex biogenesis” (Adj. *P*-val. = 1.9 × 10^−5^) in testis.

**Figure 3:**
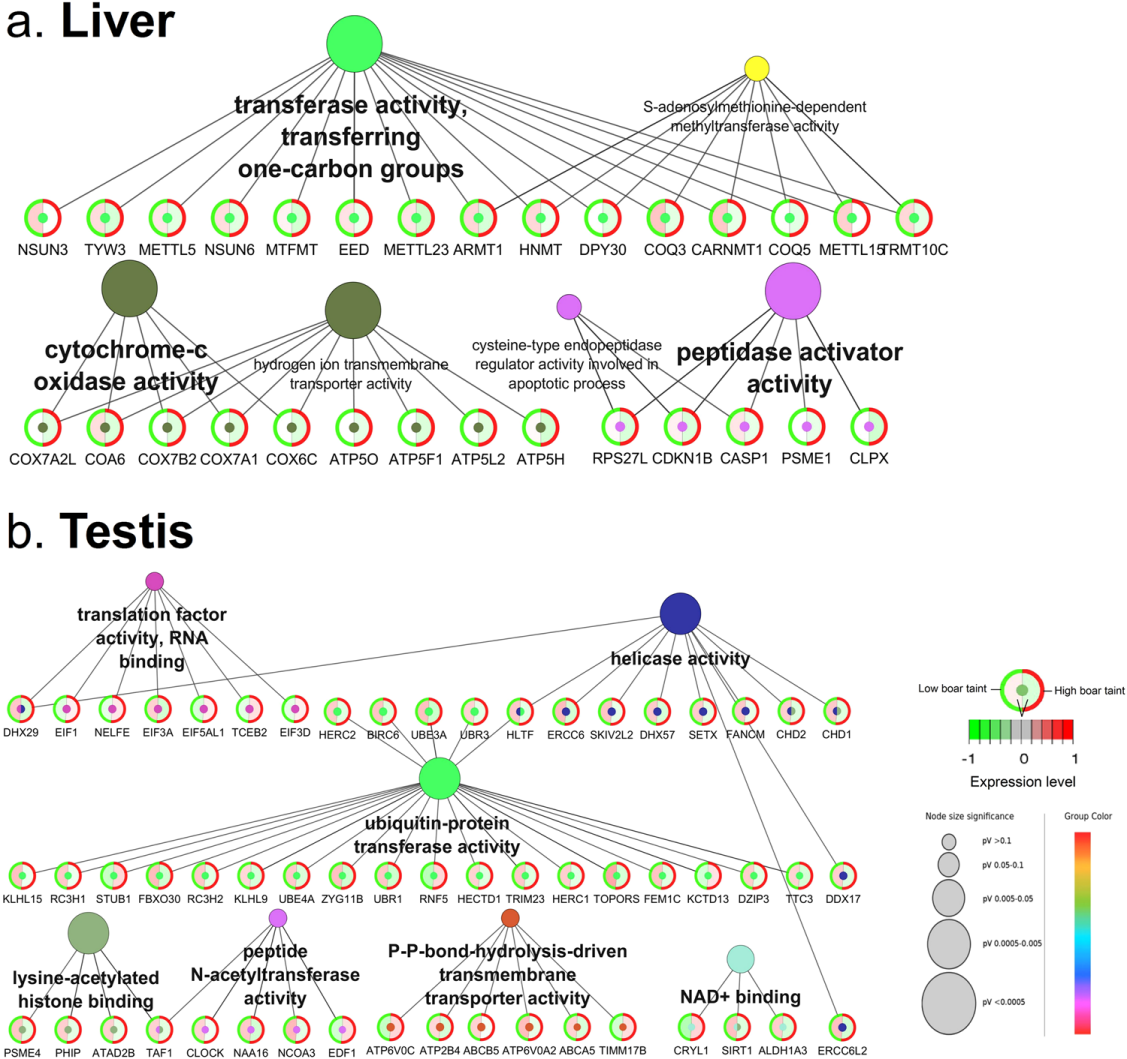
Network visualisation of molecular function gene ontology terms found in (**a**) liver and (**b**) testis obtained by from a fixed amount of the top 507 differentially expressed genes. Each term is represented by a circle whose diameter is negatively correlated with the adjusted *P* value (pV). Each node represents a gene where the expression levels are indicated in the circle where the green group (left side) represents the expression level in low BT group and the red group (right side) represents the expression level in high BT group.

Gene functional enrichment analysis of a fixed number (*n* = 507) of the top DE genes from each tissue revealed the MF GO terms “Transferase activity, transferring one-carbon groups”, “S-adenosylmethionine-dependent methyltransferase activity”, “Cytochrome-C oxidase activity” with child GO term “Hydrogen ion transmembrane transporter activity” and “Peptidase activator activity” with child GO term “Cysteine-type endopeptidase regulator activity involved in apoptotic process” significantly enriched (Adj. *P*-val. < 0.05) in liver. Furthermore, “Translation factor activity, RNA binding”, “Helicase activity”, “Ubiquitin-protein transferase activity”, “ Lysine-acetylated histone binding”, “Peptide N-acetyltransferase activity”, “P-P-bond-hydrolysis-driven transmembrane transporter activity” and “NAD + binding” were significantly enriched (Adj. *P*-val. < 0.05) in testis. The MF GO terms from both tissues were visualised with the DE genes mapped to their corresponding GO terms and the expression levels in the “low BT” and “high BT” groups for liver (Fig. 3a) and testis (Fig. 3b).

### Pathway enrichment analysis

**Figure 4:**
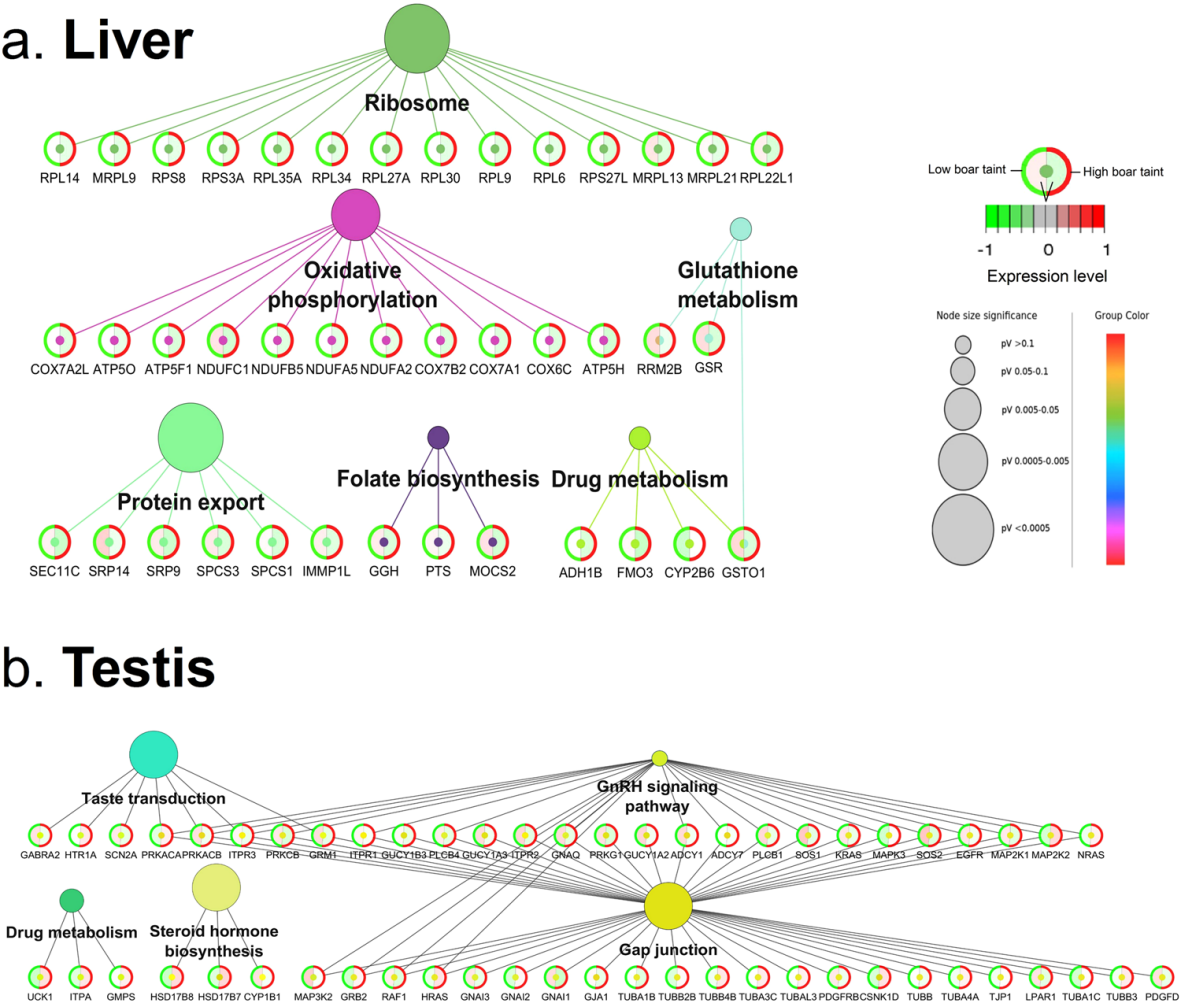
Network visualisation of a selection of the most biologically interesting and overrepresented pathways in (**a**) liver and (**b**) testis. Each pathway is represented by a circle whose diameter is negatively correlated with the adjusted *P*-value (pV) of the given term. Each node represents a gene where the expression levels are indicated in the circle where the green group (left side) represents the expression level in low BT group and the red group (right side) represents the expression level in high BT group.

Pathway analysis revealed six and 20 significantly enriched (Adj. *P*-val. < 0.05) Kyoto Encyclopedia of Genes and Genomes (KEGG) pathways in liver and testis, respectively (Supplementary file 4). The top three significantly enriched pathways found were “Ribosome” (Adj. *P*-val. = 5.0 × 10^−4^), “Protein export” (Adj. *P*-val. = 6.4 × 10^−4^) and “Alzheimer’s disease” (Adj. *P*-val. = 4.3 × 10^−3^) in liver and “Neuroactive ligand-receptor interaction” (Adj. *P*-val. = 1.1 × 10^−9^), “Cytokine-cytokine receptor interaction” (Adj. *P*-val. = 1.1 × 10^−6^) and “Thyroid hormone signalling pathway” (Adj. *P*-val. = 7.8 × 10^−5^) in testis. A selection of pathways deemed biologically interesting were visualised with their DE genes mapped to their corresponding pathways and the expression levels in the “low BT” and “high BT” in liver (Fig. 4a) and testis (Fig. 4b). These pathways were “Ribosome”, “Oxidative phosphorylation”, “Gluthathione metabolism”, “Protein export”, “Folate biosynthesis” and “Drug metabolism” in liver and “Taste transduction”, “GnRH signalling pathway”, “Drug metabolism”, “Steroid hormone biosynthesis” and “Gap junction” in testis.

### Co-expression network analysis

**Figure 5:**
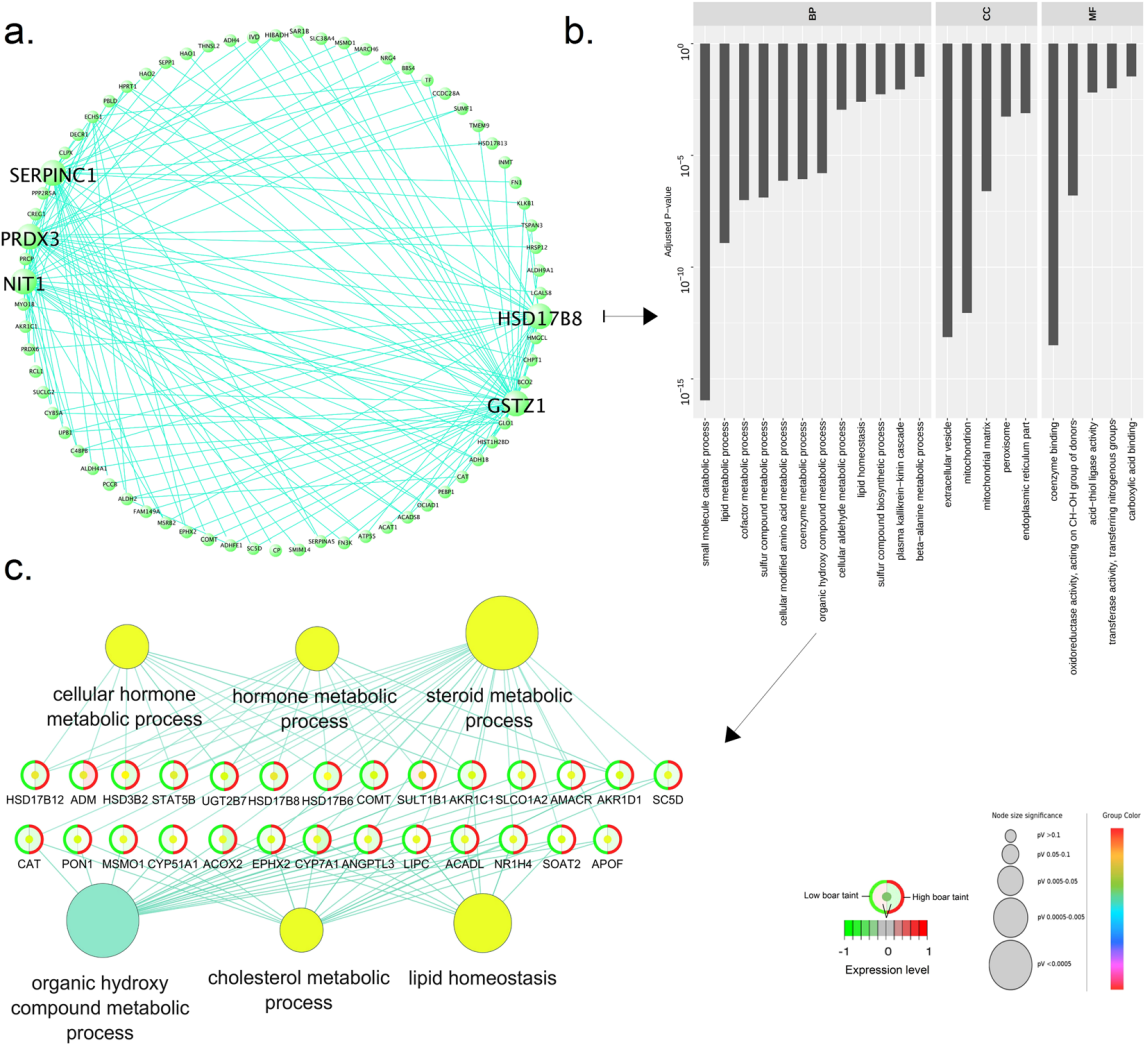
Composite visualisation of the *lightgreen* module in liver. (**a**) Network visualisation of hub genes in the module. Hub genes are indicated by large font and circle size; (**b**) Bar plot of gene ontology (GO) terms related to the module. Y-axis indicate the GO term, X-axis indicate the adjusted *P*-value and (**c**) analysis of genes related to the GO term “Organic hydroxy compound metabolic process”. Abbreviations: *BP* Biological process, *CC* cellular component, *MF* molecular function.

In liver, no modules were found to be significantly (*P* < 0.05) associated with genetic merit of BT, but one module (*lightgreen*) containing 593 genes was found to have a strong tendency of association with genetic merit of BT (*P* = 0.06) with a negative correlation (module-trait relationship (r) = −0.27). Hence, the module was selected for additional analysis. *SERPINC1*, *PRDX3*, *HSD17B8*, *GSTZ1* and *NIT1* were found to be hub genes (Fig. 5a). The intramodular connectivity defined as the node degree (*k*) (links per node) of the individual hub gene ranged from 23 to 36 and module membership measure ranged from 0.77 to 0.84. Enrichment analysis of the genes from the *lightgreen* module revealed “Small molecule catabolic process”, “Lipid metabolic process” and “Cofactor metabolic process” as the top three significantly (adj. *P*-val. < 0.05) enriched BP GO terms (Fig. 5b). Due to its biological relevance, GO term “Organic hydroxyl compound metabolic process” was selected for further analysis which revealed child GO terms of “Cellular hormone metabolic process”, “Hormone metabolic process”, “Steroid metabolic process”, “Cholesterol metabolic process” and “Lipid homeostasis” (Fig. 5c).

**Figure 6:**
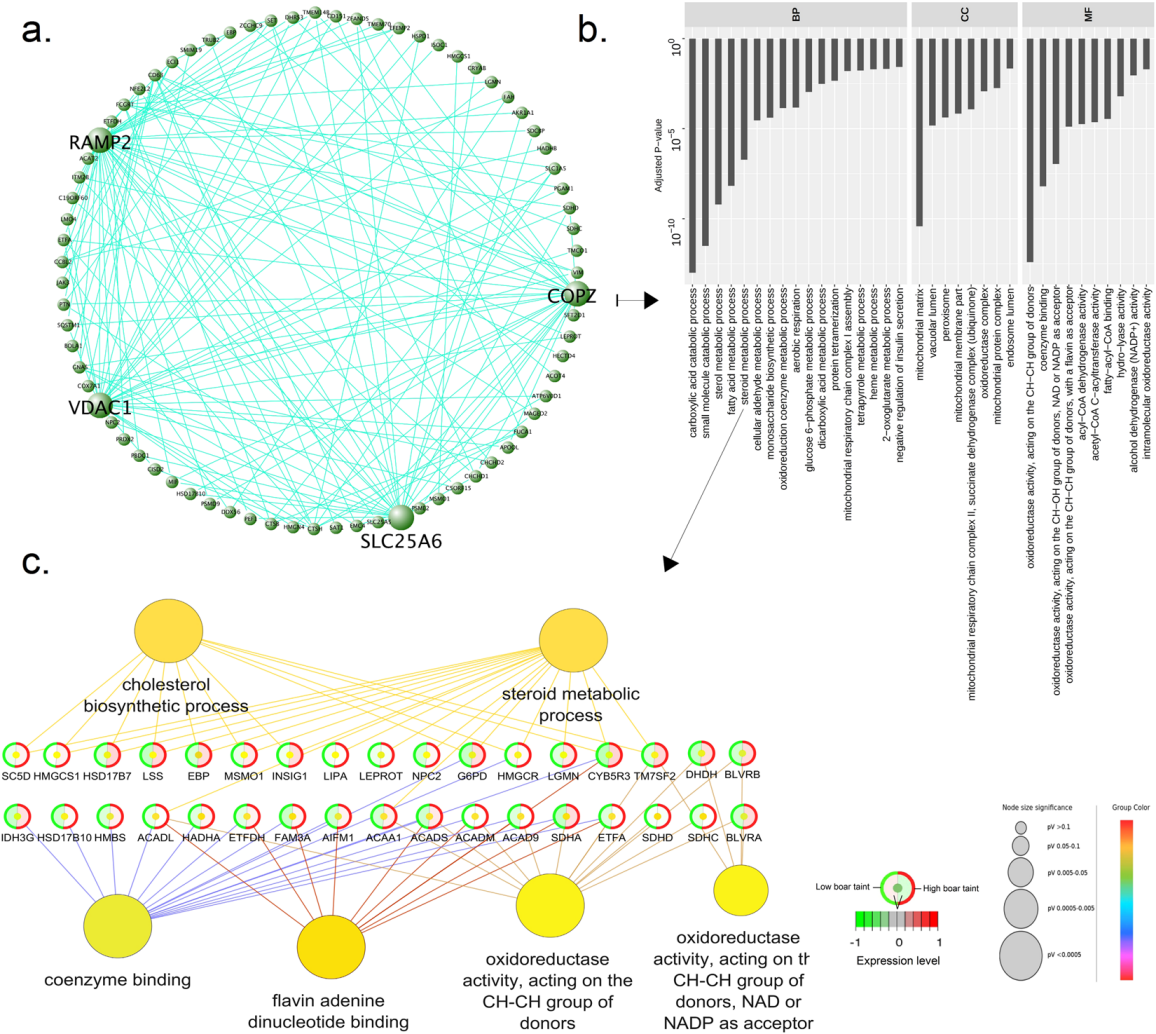
Composite visualisation of the *darkgreen* module in testis. (**a**) Network visualisation of hub genes in the module. Hub genes are indicated by large font and circle size; (**b**) Bar plot of gene ontology (GO) terms related to the module. Y-axis indicates the GO term, X-axis indicates the adjusted *P*-value and (**c**) analysis of genes related to the GO term “Steroid metabolic process”. Abbreviations: *BP* Biological process, *CC* cellular component, *MF* molecular function.

In testis, two modules were found to be significantly (*P* < 0.05) associated with genetic merit of BT: *darkgreen* and *tan* containing 180 and 519 genes, respectively. Furthermore, the *darkturquoise* module was found to have a tendency of association with genetic merit of BT (*P* = 0.05). The module *darkgreen* containing 180 genes was positively correlated with the summarised EBVs (r = 0.31), whereas *tan* had a negative correlation (r = −0.49). *COPZ2*, *RAMP2*, *VDAC1* and *SLC25A6* were found to be hub genes in the *darkgreen* module (Fig. 6a) with intramodular connectivity ranging from 24 to 35 and module membership measure ranging from 0.34 to 0.53. The top three most significantly (adj. *P*-val. < 0.05) enriched BP GO terms attributed to *darkgreen* module were “Carboxylic acid catabolic process”, “Small molecule catabolic process” and “Sterol molecule metabolic process” (Fig. 6b). The GO term “Sterol metabolic process” was selected for further analysis which revealed further GO annotations of “Cholesterol biosynthetic process”, “Coenzyme binding”, “Flavin adenine dinucleotide binding”, “Oxidoreductase activity, acting on the CH-CH group of donors”, and “Oxidoreductase activity, acting on the CH-CH group of donors, NAD or NADP as acceptor” (Fig. 6c). Full result tables from co-expression analysis are included in Supplementary file 5.

### Candidate biomarker selection

**Figure 7:**
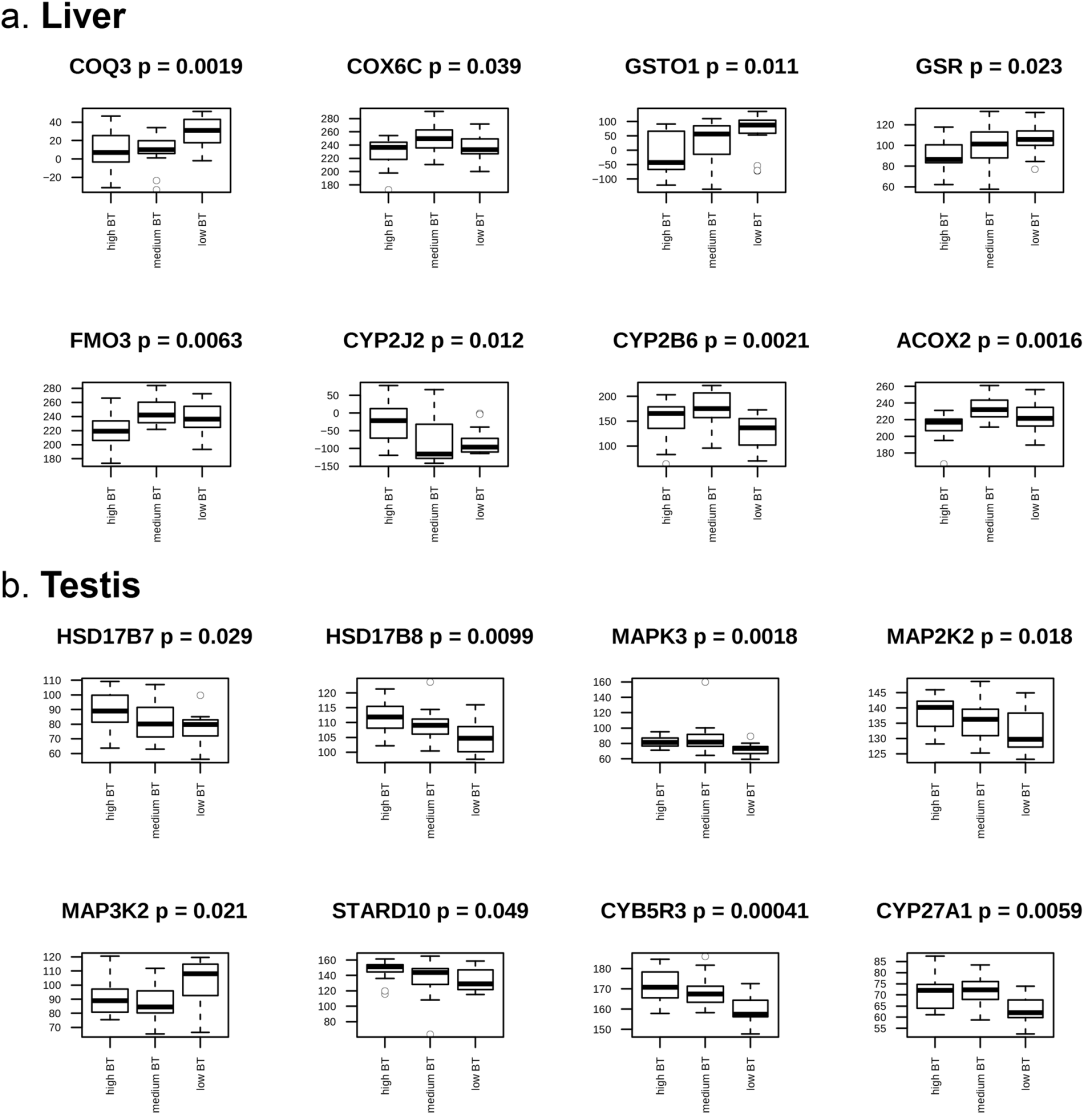
Selected candidate biomarkers with their associated box plot from (**a**) liver and (**b**) testis. Y-axis indicates normalised expression levels. X-axis indicates low, medium and high boar taint (BT) groups. Finally, the *P*-value (p) indicates the result from a separate Kruskal-Wallis test on expression levels of the candidate biomarker throughout the three BT groups.

By combining results from differential expression analysis with co-expression analysis and applying a reductionist strategy, a total of 16 candidate biomarkers were detected as potential biomarkers: *COQ3*, *COX6* *C*, *GSTO1*, *GSR*, *FMO3*, *CYP2J2*, *CYP2B6* and *ACOX2* in liver (Fig. 7a) and *HSD17B7*, *HSD17B8*, *MAPK3*, *MAP2K2*, *MAP3K2*, *STARD10*, *CYB5R3* and *CYP27A1* in testis (Fig. 7b). Briefly, the reductionist strategy comprised selection of candidate biomarkers from genes that were found as significant (FDR < 0.05) in the differential expression analysis and significant (*P* < 0.05) in additional filtering by expression profile comparison. Furthermore, the genes were either: (i) annotated to relevant GO terms and/or pathways and/or (ii) part of a co-expression module associated with genetic merit of BT.

## Discussion

In this study, candidate biomarkers associated with divergent genetic merit of boar taint (BT) was identified from RNA sequencing (RNA-Seq) of liver and testis of Danish Landrace pigs. The genetic merit of BT was defined from the estimated breeding values (EBVs) of skatole concentrations and human nose score (HNS) obtained from the sires of each pig. The HNS method has been shown to be a rapid, sensitive and cost-effective system with good correlations with androstenone and skatole levels and contains the ability to capture subjective variation not obtained by laboratory assays^44^. However, the HNS method is prone to some limitations that are intrinsically linked to using human beings as trained panellists: (i) regional differences in panels perception of BT compounds and training of panellists^45^; (ii) moderate reproducibility due to differences in human sensitivity and perception of BT even in experienced panellists^46^ and (iii) accuracy of panels is related to the number of panellists and their individual olfactory acuity^47^. The EBVs (the relative genetic performance compared with a contemporary group^43^) were calculated by Genomic BLUP Animal Models^48,49^ and is used by breeding organisations as a measure of genetic merit. This is related to the advantage of EBVs which can be calculated for a large number of animals without phenotypic data via their pedigree or genomic relationship with animals having phenotypic data by a Best Linear Unbiased Prediction (BLUP) framework. Collecting phenotypic data of BT traits on large number of animals is also very prohibitive. As this study was concerned with animal breeding under industry settings, we integrated EBVs from the breeding sires with gene expression profiles of their progeny to find candidate biomarkers associated with genetic merit of BT. Hence, biomarkers will be able to identify pigs with low genetic merit of BT for selection, before they develop BT and have the trait recorded which is of high relevance in practical and cost-effective breeding programs. The EBVs were adjusted for age (in days) and carcass weight (in kg) at slaughter. Thus, systematic differences in sexual maturation between pigs were accounted for in order to ensure consistent steroidogenic potential in the three groups, as warranted in previous research^33^. Gene expression profiles were not subjected to further correction by any covariates other than technical normalisation. Previous work found EBVs as a very reliable phenotype indicator^50^ and they have been successfully applied in gene co-expression analysis^51^.

A shortcoming of the current study is the lack of phenotypic data. Due to the commercial nature of the pigs, we did not have access to phenotypic measurements. In a previous study by Lervik, *et al*.^50^, the correlation between EBVs of androstenone and the androstenone phenotype was high. On the other hand, skatole has a slightly lower heritability than androstenone^30^ and we expect some disparity due to lack of correlation between EBVs and phenotype in the individual pig at slaughter weight (~100 kg). Thus, the lack of phenotypic data means that any disparity between genetic merit and BT phenotype cannot be identified or quantified. However, we emphasise that the current study identifies candidate biomarkers of genetic merit of BT and the candidate biomarkers found in this study cannot be used directly for phenotype identification without prior validation.

Liver metabolism of xenobiotic substances is divided into two phases: phase I metabolism which comprises oxidation, reduction and hydroxylation steps to make the substrate more soluble in water by adding or exposing a functional group and phase II sulfoconjugation which adds polar groups for increased hydrophilicity and excretion^52,53^. Related to the phase II sulfoconjougation, transferase activity was found to be highly enriched GO term in liver, but not in testis (Fig. 2a). Transferases are a large group of enzymes involved in transfer of specific functional groups from one molecule to another and previous studies have indicated their importance in catalysis of phase II sulfoconjugation of 16-androstene steroids in liver and testis^52,54^. All eleven DE genes annotated to “Single-carbon transferase activity” in liver (Fig. 3a) were downregulated in high BT pigs, indicating a strong decrease in phase II metabolism of BT compounds. Furthermore, transferase activity was found enriched in both tissues which emphasises the importance of transferase-related genes of BT. Interestingly, *COQ3* encodes an O-methyltransferase that is required for two steps in the biosynthetic pathway of coenzyme Q 10 (CoQ10). CoQ10 is involved in the respiratory chain and participates in aerobic cellular respiration which generates energy in the form of ATP^55^. In mammals, tissues with high energy demands, such as heart, liver and kidney, have the highest CoQ10 concentrations^56,57^. As the BT condition is dependent on the level of liver degradation, it is likely that the downregulated expression of *COQ3* reflects insufficient metabolism. Hence, *COQ3* is considered a candidate biomarker for BT in liver (Fig. 7a).

Important enriched MF terms in liver included “cytochrome-c oxidase activity” which comprised isoforms of *COX6* and *COX7*. These genes encode subunits of cytochrome-c oxidase, the terminal enzyme of the mitochondrial respiratory chain catalysing the electron transfer from reduced cytochrome-c to oxygen^58^. Previous studies reported genes encoding cytochrome oxidases of the mitochondrial respiratory chain to be associated with skatole levels^12,59–62^. Specifically, the cytochrome subunit family *COX4I1* and *COX8 C* encoding cytochrome-c oxidases were involved in regulation of skatole metabolism^63,64^, which was attributed to their involvement in the phase I oxidation reactions in liver. In this study, the cytochrome-c oxidases *COX6* *C*, *COX7AL*, *COX7B2* and *COX7A1* were all downregulated in the high BT pigs which may reflect the situation of deficient skatole metabolism in high BT pigs. Another strong indicator of the importance of cytochrome-c oxidases in the context of BT is the high enrichment of mitochondrial CC GO terms where the enzymes are located. Hence, *COX6* *C* is considered a candidate biomarker for BT in liver (Fig. 7a). Another important MF term found enriched in liver is the oxidoreductases, specifically the “CH-CH oxidoreductases” which participate in the phase I metabolism of BT compounds and play important enzymatic roles, some which have not yet been precisely clarified^65^. In this study, the “CH-CH oxidoreductases” comprised *ACAD10*, *ACADSB*, *ACOX2*, *CPOX* and *DUS4L* (Supplementary file 3) which are commonly found in gene expression studies for BT in liver^65^ and testis^66,67^. Interestingly, all five genes were downregulated in the high BT pigs (Supplementary file 2), which may be attributed to the insufficient liver degradation of BT compounds. *ACOX2* encodes a protein of the acyl-Coenzyme A oxidase family which involves degradation of long branched fatty acids and bile acid intermediates in peroxisomes^68^. The similar *ACOX1* was previously found to be slightly upregulated in high BT pigs^65^. The current finding suggests a contrary expression pattern for *ACOX2* as the gene is indeed downregulated in high BT pigs. *ACOX2* is also found in the *lightgreen* CE module in liver where the gene is functionally annotated to “Steroid metabolic process” (Fig. 5c). Hence, *ACOX2* is considered a candidate biomarker for BT (Fig. 7a).

Analysis of overrepresented pathways in liver revealed a strong correlation towards active transcription (“Ribosome”), export of proteins (“Protein export”) and oxidation (“Oxidative phosphorylation”) (Fig. 4a). Analysis of the specific DE genes annotated to “Drug metabolism” revealed *FMO3* and *CYP2B6*. Flavin-containing monooxygenases (FMOs) are a family of enzymes that converts lipophilic compounds into polar metabolites, thus decreasing their activity^69^. The FMOs are involved in metabolic activation or detoxification of numerous xenobiotics such as pesticides, drugs and carcinogens^70^. FMOs are regulated by sex steroid hormones and *FMO3* was found to be repressed by testosterone and upregulated by castration^71^. Furthermore, *FMO3* was previously speculated to be associated with off flavour in pork^72^. Consistently, we found *FMO3* downregulated in high BT as androstenone and testosterone levels are positively correlated^7^. Hence, *FMO3* is considered a candidate biomarker for BT in liver (Fig. 7a). The cytochrome P450 (CYP) proteins are also monooxygenases and similar in function to FMOs; they are involved in drug metabolism and synthesis of cholesterol, steroids and other lipids^73^. In human and pig, 18 families of CYPs have been identified with 57 and 54 genes, respectively^73^. The CYPs have previously been reviewed as candidate genes for BT^37^. In the current study, ten CYPs were identified as DE genes: *CYP2J2*, *CYP2B6* in liver and *CYP4V2*, *CYP1B1*, *CYP2D7*, *CYP2R1*, *CYP51A1*, *CYP20A1*, *CYP26B1* and *CYP27A1* in testis (Supplementary file 2). In liver, *CYP2B6* was upregulated in the high BT group which clearly reflects its implication in phase I metabolism. Another member of the *CYP2* family was the *CYP2J2* which was found to be upregulated in high BT. Interestingly, previous studies showed that the *CYP2B* was found only to metabolise skatole to a low degree and to our knowledge, no data is available for *CYP2J* (see a review by Rasmussen and Zamaratskaia^74^). In testis, *CYP27A1* was found to be strongly upregulated in high BT pigs. In porcine hepatocytes, *CYP27A1* has been found to be active in 25-hydroxylation of the prohormone vitamin D_3_ and to carry out the 27-hydroxylation of cholesterol-derived intermediates in bile acid biosynthesis^75^. In this context, it is likely that *CYP27A1* is affected by the increased steroid production characterised in high BT pigs and to our knowledge, we are the first to report association of the gene with BT. Hence, *CYP2J2*, *CYP2B6* and *CYP27A1* are considered candidate biomarkers for BT in liver and testis, respectively (Fig. 7).

*GSTO1* and *GSR* are part of “Glutathione metabolism” and “Drug metabolism” pathways and participate in the phase II metabolism. *GSTO1* encodes a member of the glutathione S-transferases (GSTs) that are known for their role in catalysis of conjugation reactions in endogenous substances, haeme, fatty acids, xenobiotics and products of oxidative processes^76^. In context of BT, *GSTO2* has been speculated to be involved in excretion of skatole from the porcine body^77^. We found *GSTO1* to be clearly downregulated in high BT pigs (Fig. 4a), which could explain the BT condition observed by decreased excretion of skatole. The finding is consistent with previous gene expression studies in liver^65^ and testis^66^. Due to its implication in relevant pathways and clear expression pattern, *GSTO1* could be considered a candidate biomarker (Fig. 7a). *GSR* encodes a glutathione reductase enzyme that catalyses the reduction of glutathione disulphide to glutathione, an important antioxidant and critical for the cell to resist oxidative stress and to maintain its reducing environment^78^. The expression of *GSR* is probably related to liver degradation capacity as *GSR* exhibited a clear pattern of downregulation in high BT pigs. Hence, *GSR* is considered a candidate biomarker in liver (Fig. 7a). However, the precise relationship between *GSR* and BT compounds should be subject to further research.

Analysis of DE genes from testis revealed “Transmembrane signalling receptor activity” to be a highly enriched MF GO term. This may reflect the fact that steroid hormone biosynthesis is a highly active function in the testis of high BT pigs, as previous research has found a high genetic correlation between androstenone (plasma and fat) and sex steroids in Duroc and Landrace pigs^79^. Activation and regulation of steroid hormone biosynthesis requires a high level of cellular communication observed by enrichment of the MF GO terms “Poly(A) RNA binding” and “Enzyme binding” which reflects the high pathway activity. Furthermore, the CC GO terms such as “Intracellular part” and “Nuclear part” revealed that most processes are located in the intracellular part or nuclear part where transcription and translation are undertaken in the cell (Fig. 2b). The BP term “Organelle organization” was highly enriched which probably reflects high activity of transcription and protein metabolic processes. When a fixed number of top DE genes between tissues were subjected to MF GO term enrichment analysis, “Translation factor activity, RNA binding” had four upregulated genes in high BT pigs which probably is attributed to the high level of steroidgenesis. Interestingly, genes associated with the term “Ubiquitin-protein transferase activity” were almost exclusively downregulated in the high BT pigs. This family of transferases is responsible for the transfer of ubiquitin from one protein to another which can signal the degradation of the protein by the proteasome. The downregulation of the associated genes implies a markedly less regulated steroidgenesis which probably results in overproduction of steroid hormones in testis. This hypothesis is supported by the significant overrepresentation of the pathway “Steroid hormone biosynthesis” which comprises the 17 β-hydroxysteroid dehydrogenase (HSD) genes *HSD17B7* and *HSD17B8* (Fig. 4b). The *17β-HSD* encode enzymes that are part of the last catalysis step in the formation of androgens and estrogens^80^ and the various types provide specific cell types with the mechanisms to regulate intracellular androgens and/or estrogens^81^. The importance of the *HSD* family in relation to BT compounds has been well documented in both RNA-Seq and microarray studies and both *HSD17B2* and *HSD17B4* have previously been suggested as biomarkers for reduced BT^37,66,77,82^. We also found the *HSD17B7* and *HSD17B8* to be highly upregulated in the high BT pigs. *HSD17B7* is involved in biosynthesis of cholesterol^83^ and sex steroids^84^ and regulates bioavailability of both androgens and estrogens, yet interestingly, previous work has confirmed that the *HSD17B7* is not the key enzyme responsible for androstenone and testosterone metabolism in porcine hepatocyte^85^. However, we found the *HSD17B7* upregulated in testis and not liver, which consequently justifies the proposition of it being a candidate biomarker in testis (Fig. 7b). The *HSD17B8* is involved in regulation of concentrations of biologically active estrogens and androgens^86^. Recently, the protein encoded by the *HSD17B8* was identified as a major interaction partner in gene networks of pigs with divergent androstenone levels. The major function of the protein was hypothesised to be testosterone inactivation^87^. The testicular testosterone concentrations were positively correlated with androstenone concentrations due to active testosterone synthesis which could also imply active androstenone synthesis^87^. However, we found that *HSD17B8* was upregulated in testis from high BT pigs which is contrary to the hypothesis that *HSD17B8* inactivates testosterone synthesis. We suggest that the gene plays an important role in BT and is considered a candidate biomarker, but further research should clarify its exact role in regulation of BT compounds (Fig. 7b).

The selection of pathways of biological interest included the “Drug metabolism”, “Estrogen signalling pathway” and “GnRH signalling pathway” (Fig. 4b). In “GnRH signalling pathway”, genes had both up- or downregulation patterns, but mostly downregulated. *MAPK3* and *MAP2K2* were upregulated whereas *MAP3K2* were downregulated in high BT pigs, respectively (Fig. 4b). The MAPK pathway encodes protein kinases which have been shown to modulate numerous male reproductive functions such as spermatogenesis, sperm maturation and activation and other functions of the germ cells^88^. Interestingly, *MAPK3*, *MAP2K2* and *MAP3K2* were also a part of the “Estrogen signalling pathway” (Supplementary file 4). The “Estrogen signalling pathway” was significantly overrepresented, which is probably due to secretion of large amounts of estrogen from the testis^89^. Estrogen concentrations have been found to be correlated with androstenone in adipose tissue^90,91^. The roles of the MAPK pathway in variation of BT has recently been shown in a gene expression study on Duroc boars^92^. The authors found that upregulation of *MAPK* coincided with pubertal increase in androstenone and testosterone production^92^ as the upregulation was observed several weeks earlier than complex morphological restructuring of testis into reproductive tissue. Furthermore, proteins encoded by *MAPK* may regulate the Steroidogenic Acute Regulatory protein (STAR) (a cholesterol traffic regulator) and have a complex interaction with steroidgenesis (see review by Manna and Stocco^93^). Interestingly, *STARD10*, which may play a metabolic role in sperm maturation and lipid transport^93^, was found upregulated in testis (Supplementary file 2) and may have other localised functions such as enrichment of lipids in milk in the mammary glands^94^. Previous works have documented the role of a subfamily of *STAR* in BT in testis, especially *STARD6* and *STAR* ^66,92^. To our knowledge this is the first time a relationship between *STARD10* and BT has been reported and the association warrants further investigation of the role of *STARD10* in BT. Due to the complexity of *MAPK* in BT and the warrant for further research of these genes, *MAPK3*, *MAP2K2*, *MAP3K2* and *STARD10* are considered candidate biomarkers (Fig. 7b).

Co-expression analysis in liver revealed the *lightgreen* module to be associated and slightly downregulated with the summarised EBVs. Among the five hubs genes (*HSD17B8*, *GSTZ1*, *SERPINC1*, *PRDX3 and NIT1*), *HSD17B8* was also DE in testis and selected as a candidate biomarker. Furthermore, *HSD17B8* was co-expressed with *CYB5A*. Expression levels of *CYB5A* has previously been linked to androstenone levels^94^. Hence, the finding of *HSD17B8* as a hub gene strengthens the hypothesis of a regulatory role in androstenone synthesis. *GSTZ1* was found as a hub gene in the *lightgreen* module and as a DE gene in liver which encodes a member of the GSTs^95^. Other hub genes included *SERPINC1* (Fig. 5a) which encode a gene that inhibits thrombin as well as other activated serine proteases of the coagulation system and regulates the blood coagulation cascade^96^. Members of the *SERPIN* family have previously been associated with skatole metabolism in liver^77^ and it is very likely that the hub genes are all related through various mechanisms in the clearing of skatole and/or androstenone from the liver either directly or indirectly through regulation or activation of other genes and/or pathways. Interestingly, *SERPINC1* was co-expressed with *HSD17B13* which is not exactly clear as 17β-HSD catalyses biosynthesis of androgens and estrogens but the finding warrants further research into the association of *SERPINC1* and BT. The *darkgreen* module was significantly enriched for the MF GO term “Steroid metabolic process” which comprised *SULT1B1* and *CYP7A1*. Downregulation of *CYP7A1* has been speculated to increase androstenone levels through reduced fat catabolism^82^ which is consistent with the current study where the gene was downregulated in high BT pigs. Considering the finding of *CYP7A1* as co-expressed in liver, additional research should be focused on its role in BT. *SULT1B1* encodes a member of the sulfotransferases which catalyse the sulphate conjugation of steroid hormones, neurotransmitters, drugs and xenobiotic compounds^97^ and previous work established them as key enzymes involved in the testicular and hepatic metabolism of androstenone^66^. The closely related *SULT1A1* were found to be involved in phase II metabolism of skatole^98^ and a mutation in the gene is thought to be responsible for skatole accumulation^99^ which has prompted researchers to consider it a candidate biomarker for BT^37^. Indeed, the sulfotransferases seem important in the metabolism of BT compounds as a negative correlation between accumulation of androstenone and *SULT2A1* in which low activity of *SULT2A1* was found in animals with high concentrations of androstenone^100^. Similar results were found in a study performed on Duroc, Norwegian Landrace and Yorkshire^67^ which is also consistent with the findings in the current study where *SULT1B1* was downregulated in high BT pigs. The finding of *SULT1B1* as a co-expressed gene reflects a possible regulatory association and warrants further research on its precise role in BT. In the current study, only *SULT4A1* was found as a DE gene in testis and upregulated in high BT pigs but it did not pass additional filtering (Supplementary file 2).

In testis, the significantly associated *darkgreen* module contained genes significantly enriched for “Carboxylic acid catabolic process”, “Small molecule catabolic process” and “Sterol molecule metabolic process” (Fig. 6b). Further analysis of the “Sterol metabolic process” term revealed that several genes overlapped with “Cholesterol biosynthetic process” (Fig. 6c). Interestingly, the previously selected candidate biomarker *HSD17B7* was part of the *darkgreen* module and the finding of *HSD17B7* as a co-expressed gene may point to some kind of regulatory function in BT. However, this is in contrast to previous research where *HSD17B7* was excluded as a key enzyme responsible for androstenone and testosterone metabolism in porcine liver cells^85^. Another important enriched gene in the module was *CYB5R3* which encodes a cytochrome b5 reductase^101^. The subfamily *CYB5* has been found particular important in androstenone biosynthesis and expression levels and a mutation in *CYB5A* was speculated to decrease androstenone levels^94,102^. From its role as a reductase, *CYB5R3* is a modulator of the activity of *CYB5A* ^103^. Interestingly, work on nuclear receptor transactivation found that *CYB5R1* probably increased levels of 16-androstene steroids but ambiguous results were found for *CYB5R3* ^104^. In the current study, *CYB5R1* was also found as a DE gene in testis (Supplementary file 3) but did not pass additional filtering. Due to strong upregulation and co-expression of *CYB5R3* in high BT pigs, we consider the gene as a candidate biomarker in testis (Fig. 7b).

In this study, the candidate biomarkers were genes with functions within phase I (*COQ3*, *COX6 C*, *CYP2J2*, *CYP2B6*, *ACOX2*) and phase II metabolism (*GSTO1*, *GSR*, *FMO3*) of skatole and androstenone in liver to steroidgenesis (*HSD17B7*, *HSD17B8*, *CYP27A1*), regulation of steroidgenesis (*STARD10*, *CYB5R3*) and GnRH signalling (*MAPK3*, *MAP2K2*, *MAP3K2*) in testis. For comparison, a recently published review by Zadinová, *et al*.^37^ summarised genes currently believed to be controlling levels of androstenone (*CYP11A1*, *CYP17A*, *CYP21A2*, *CYB5A*, *LHB*, *HSD17B7*), androstenone metabolism (*HSD3B*, *SULT2A1*, *SULT2B1*, *TEAD3*), phase I skatole metabolism (*CYP2A6*, *CYP2E1*, *CYB5A*) and phase II skatole metabolism (*SULT1A1*). Other important genes were *FMO3* ^72^, *GSTO2* ^77^, *COX4I1* ^64^, *MAPK14* ^79^, *STARD6* ^92^ and *ACOX1* ^77^. Thus, a high level of consistency is evident between known gene families involved in BT and the selection of candidate biomarkers in this study. Importantly, our study presents two new genes (*COQ3*, *GSR*) and novel gene members and isoforms of BT which is probably attributed to the use of deep sequencing of ~33 mio reads per sample to register even small fluctuations in gene expression. Some candidate biomarkers probably share similar functions with known genes of BT such as the case of *HSD3B* and *HSD17B* ^91^ or may be involved in regulation of known candidate biomarkers such as *CYB5R3* that regulates *CYB5A* ^103^. Furthermore, co-expression network analysis provided valuable insight into the complexity of both DE and non-DE genes and their functions in BT such as *CYP7A1* and *SULT1B1* and enabled discovery of hub genes with a possible regulatory association such as *HSD17B8* and *SERPINC1* co-expressed with *CYB5A* and *HSD17B13*¸ respectively.

Future work should focus on the validation of the detected biomarkers at the population-wise level as well as in other breeds to develop a sensitive and specific industry-targeted test for optimised genomic-based animal breeding to reduce BT. Gene expression data should be combined with genotypes to find expression quantitative trait loci (eQTLs) of BT and/or further utilise the power of the RNA-Seq data with additional analysis of splicing events, polymorphisms and different isoforms of transcripts^105^ of the candidate biomarkers proposed in this study.

## Methods

A complete overview of the experimental design is presented in Fig. 1.

### Animal model

Male Landrace pigs (*n* = 114) were housed at the commercial testing station Bøgildgård in Denmark (N 56°27′52.60′′ W 9°38′62.97′′) operated by the Pig Research Centre, SEGES (Copenhagen, Denmark) and raised with *ad libitum* feed and water supply. The pigs were produced from sires with known genetic merit of boar taint (BT) assessed from estimated breeding values (EBVs) of skatole concentrations and human nose score (HNS). To account for both traits, the genetic merit of BT was defined as the sum of the EBVs of skatole concentration and HNS obtained from the respective sire of each pig and denoted as the summarised EBV following equation (1):

1 $${Summarised}\,{EB}{{V}}_{ij}={Skatole}\,{EB}{{V}}_{ij}+{Human}\,{nose}\,{score}\,{EB}{{V}}_{ij}$$

where the summarised EBV_*i*_ is the sum of the EBV for skatole concentration in fat and the EBV for HNS for the individual pig *i* obtained from its respective sire *j*. All EBVs were collected from the Danish pig breeding database (Pig Research Centre, SEGES, Copenhagen, Denmark). The raw values for skatole concentrations were obtained from measurements on carcass fat samples by a calorimetric method^106^. HNS were collected by a standardised method^107^. The EBVs (the relative genetic performance compared with a contemporary group) were calculated by Genomic BLUP Animal Models^48,49^. The EBVs were corrected for age (days) and weight (kg) in order to account for systematic differences in sexual maturation and to obtain full steroidogenic potential^33^ in the three groups.

Subsequently, the animals were grouped as low, medium or high genetic merit of BT, according to the summarised EBVs. A total of 48 pigs out of the 114 pigs were selected for analysis from each of the three groups as high, medium and low genetic merit of BT (Supplementary file 1). The selection of pigs was based on the most extreme values within each of the low and high groups, and the closest values to the mean in the medium group. To account for any pedigree effects, the maximum amount of relatedness allowed between pigs but within sire groups were only half-siblings. However, the sires themselves were not related as far as we could find in the dataset.

### Tissue collection and preparation

Pigs were slaughtered at a weight of ~100 kg at a commercial slaughterhouse (Danish Crown, Herning, Denmark). Slaughter was performed by submersion into CO_2_ until unconsciousness ensued followed by exsanguination. Following slaughter, the liver was extracted from the carcass and 150 mg of tissue were retrieved by punch biopsy and immediately immersed into 1.5 ml RNAlater (QIAGEN, Hilden, Germany) in 2 ml Eppendorf tubes (Eppendorf, Hamburg, Germany). The carcasses were kept in a cold room at 4 °C for approximately 1.5 h before 150 mg testis tissue were retrieved by punch biopsy and immersed into 1.5 ml RNAlater (QIAGEN). All samples (*n* = 96) were stored at −20 °C for 14 days until RNA extraction and sequencing at a commercial facility (AROS A/S, Aarhus, Denmark).

### RNA extraction and sequencing

Total RNA was extracted from the 96 samples by RNeasy Mini Kit (QIAGEN) following instructions of the manufacturer. Concentration of RNA was measured by Nanodrop^®^ 2000 (ThermoScientific, Massachusetts, USA). The quality of the RNA was measured with a Bioanalyzer (Agilent, California, USA) to ensure an RNA integrity number (RIN) of at least 6; otherwise, the samples were discarded. For samples with an RIN values of 7 or above, the sequencing libraries were prepared using the TruSeq stranded mRNA (Illumina, San Diego, USA) kit following instructions of the manufacturer. Samples with RIN values from 6 to 7 were therefore prepared using the TruSeq total stranded RNA (Illumina) kit following instructions of the manufacturer. In both protocols, 400 ng of total RNA were used as input and fragmentation was performed following instructions of the manufacturer, but with an incubation step of 1 min at 94 °C protocol to produce libraries that are compatible with sequencing on a paired-end 100 base pair flow cell (PE-100bp FC, Illumina, San Diego, USA). Libraries were subjected to quality control with respect to size profile by test on an Agilent DNA 1000 (Agilent, California, USA) and library concentration by KAPA quantitative PCR (qPCR) kit and three independent 10^6^-fold dilutions of libraries following instructions of the manufacturer (Kapa Biosystems, Massachusetts, USA). The samples were sequenced with an Illumina HiSeq. 2500 (Illumina, San Diego, USA) which amounted to a theoretical 40 million reads per sample.

### Quality control and gene counting

Data from sequencing machine were converted from base calls into FastQ-files by CASAVA^®^-software (Illumina, San Diego, USA). Quality control (QC) of RNA-Seq reads was conducted with FastQC^108^. Reads were trimmed for known Illumina TruSeq adapter sequences using the programme CutAdapt^109^. Poor reads were trimmed by Trimmomatic^110^ using default parameters. The trimmed reads were then mapped to the *Sus scrofa* reference genome (10.2, version 79) obtained from Ensembl^111^ by the STAR aligner using default parameters^112^. Post-mapping QC was performed with Qualimap^113^. The mapped reads were counted to each gene by HTSeq count using default parameters^114^. All subsequent statistical analysis were performed in R version 3.1.0^115^. Only genes with a mean count of more than five were included in the gene count matrices. Due to relatively poor annotation of the *Sus scrofa* genome, Ensembl identifiers were translated into their human orthologous gene symbols by the R package biomaRt^116,117^, using the *Homo sapiens* reference genome (h38, version 84, Ensembl).

### Differential expression analysis

Normalisation of gene counts was performed by voom variance-stabilization function with sample quality weights^118^ implemented in the R package limma. Differentially expressed (DE) genes were detected using the R package limma^119^. Briefly, limma fits a linear model to each gene using the function lmFit following equation (2):

2 $${\gamma }_{ij}={\beta }_{j,BT}BT+{\varepsilon }_{ij}$$

where ${\gamma }_{ij}$ is the measured expression level of gene *j* for individual pig *i*, ${\beta }_{j,BT}$ is the estimated regression coefficient calculated by regression of the gene expression value of the *i* ^*th*^ individual pig categorised to its genetic merit of BT group (“low”, “medium” or “high”) for the *j* ^*th*^ gene and *ε*_*ij*_ is the error component. By using the function eBayes in limma, the moderated *t*-test statistics for differences in the variance of gene expression of a gene across replicates were calculated and subjected to multiple testing correction of the *P* values by the Benjamini-Hochberg procedure^120^ which resulted in a false discovery rate (FDR) and the logarithm-transformed fold change for each gene. Genes were categorised as differentially expressed genes when FDR was below 0.05 and were found by contrasting “high BT *vs* low BT” groups from a design matrix that included all three groups. Additional filtering was performed by comparison of expression profiles of the individual DE gene throughout the three groups by a Kruskal-Wallis test. Gene functional enrichment analysis was performed with the Cytoscape^121^ plug-in ClueGO version 2.2.5^122^. By providing DE genes and genes attributed to co-expression modules with significant association to the summarised EBVs, ClueGO performed overrepresentation test and visualisation for gene ontology (GO) terms and KEGG pathways using default parameters. Test results were subjected to multiple testing correction of the *P* values by Bonferroni correction. For semantic filtering of redundant GO terms, the online tool REVIGO^123^ was used. As the BT condition involves numerous enzymatic functions and for the sake of simplicity, the most interesting molecular function (MF) GO terms from a fixed number (*n = *507) of top DE genes from each tissue to make analysis consistent. Similar DE analyses based on 3 or more categories and treatment groups with similar sample sizes / biological replicates and RNA-Seq data in animals have been applied for various conditions including Mazzoni, *et al*.^34^ for cattle reproduction and Salleh, *et al*.^35^ for cattle feed efficiency.

### Co-expression analysis

In order to identify strongly co-expressed (CE) genes (“hub genes”) which may have regulatory functions and/or major impact on the trait, we employed a co-expression analysis approach for each tissue which has previously been successfully employed by our group^40^. Scale-free undirected co-expression networks were built using the R package WGCNA^42,124^ and modules with significant association (*P* < 0.05) to genetic merit of BT were detected and analysed. The genetic merit of BT was inputted as the high, medium and low groups defined from the summarised EBVs as described in the animal model section. The co-expression analyses was performed independently for liver and testis by the following procedure: An adjacency matrix comprising Pearson’s correlations between expression levels of genes was created from the 7,500 most highly expressed genes from the tissue in question which satisfied the requirements of both acceptable computational time and biological meaningfulness. Subsequently, the matrix was raised by a power (β) of 10 in each tissue which was found to be an appropriate value by the function pickSoftThreshold() to reach a scale-free topology index (*R* ^2^) of at least 0.90. The topological overlap measure (TOM), which assesses the degree of shared neighbours between pairs of genes, was calculated from the adjacency matrix and used to build a gene dendrogram which detects modules and assigns colour by the DynamicTreeCut algorithm^125^, using a minimum of 30 genes per module. The module eigengene, the first principal component of each module, represents the expression value of each module and was used to detect biologically relevant modules. The module-trait relationship was calculated as the Pearson’s correlation between the module eigengene and the traits of interest. Modules significantly (*P < *0.05) associated with genetic merit of BT were exported for topological analysis and visualisation in the software VisANT^126^. An arbitrarily chosen subset of 150 gene pairs with the highest TOM within the selected modules was selected and hub genes were defined as genes with highest intramodular connectivity. The intramodular connectivity was assessed by a chosen topological analysis of the module: genes with a node degree distribution (*k*) (links per node) of ≥12 were assigned as hub genes for the specific tissue.

### Candidate biomarkers

Selection of candidate biomarkers for each tissue was made by applying a reductionist strategy of genes that had significant (FDR < 0.05) differential expression and significant (*P < *0.05) difference in expression profiles by a Kruskal-Wallis test throughout the three groups and were either: (i) annotated to relevant GO terms and/or pathways and/or (ii) part of a co-expression module related to a summarised EBVs of skatole and HNS. Each chosen candidate biomarker was visualised on a box plot with the *P* value of the Kruskal-Wallis test.

## Declarations

### Ethics approval and consent to participate

Animal Care and Use Committee approval was not obtained for this study, because tissue samples were obtained from a commercial slaughter facility.

### Availability of data and material

The datasets generated during and/or analysed during the current study are available in NCBI’s Gene Expression Omnibus^127^ and are accessible through GEO Series accession number GSE93734 (https://www.ncbi.nlm.nih.gov/geo/query/acc.cgi?acc=GSE93734).

All data analysed during this study are included in this published article [and its supplementary information files].

## Electronic supplementary material

Dataset 1

Dataset 2

Dataset 3

Dataset 4

Dataset 5

Supplementary information
