# Supplementary material for: Differential expression and co-expression gene networks reveal candidate biomarkers of boar taint in non-castrated pigs
Source: Sci Rep. 2017 Sep 22;7:12205. doi: 10.1038/s41598-017-11928-0 (PMC5610188; doi:10.1038/s41598-017-11928-0)
Supplement: Supplementary file 6 — Supplementary information [file 41598_2017_11928_MOESM6_ESM.pdf]

Differential expression and co-expression gene networks reveal candidate biomarkers of boar taint in non-castrated pigs

Markus Drag[1], Ruta Skinkyté-Juskiené[1], Duy N Do[2], Lisette JA Kogelman[1,3], Haja N Kadarmideen[1,4*]

Supplementary file 1: Estimated breeding values (EBVs) of human nose score (SIRE_HNS_BV), skatole concentration (SIRE_SKA_BV) and the two traits summarised to one trait (total) for all individual pigs in the study. Each pig was assigned to low, medium or high boar taint (condition), according to its "total" value.

Supplementary file 2: Differentially expressed genes in liver and testis. Tissue column indicates liver or testis. Each gene is identified by its Ensembl gene ID, its gene symbol, a description, the log-fold change (logFC), the average expression (AveExp), t statistic (t), *P* value (P.Value), adjusted *P* value (adj.P.Val), beta-statistic (B) and the *P*-value by comparison of expression profiles by Kruskal-Wallis test (Kruskal-Wallis). IDs and description were obtained from the R-package *biomaRt*. (http://www.ensembl.org), while statistical results were obtained from the R-package *limma*.

Supplementary file 3: Gene ontology (GO) terms obtained from differentially expressed genes from liver and testis. Tissue column indicates liver or testis. Each GO term is identified by its id (GO:ID), name (GO_term), ontology source and version (Ontology Source), *P*-value (term PValue), adjusted *P*-value (Term PValue Corrected with Bonferroni step down), number of associated genes (% Associated

Genes), number of genes (Nr Genes) and the genes associated to each pathway (Associated Genes Found).

Supplementary file 4: Kyoto Encyclopedia of Genes and Genomes pathways for liver and testis with their associated genes. Tissue column indicates liver or testis. Each pathway is identified by its id (GO:ID), name (KEGG_Pathway), ontology source and version (Ontology Source), *P*-value (term PValue), adjusted *P*-value (Term PValue Corrected with Bonferroni step down), number of associated genes (% Associated Genes), number of genes (Nr Genes) and the genes associated to each pathway (Associated Genes Found).

Supplementary file 5: Raw output from WGCNA analysis of liver and testis gene expression profiles. Columns indicate: tissue where the results were obtained from (Tissue), name of each gene (geneSymbol), assigned module to each gene (moduleColor), total gene significance value (GS.total), total gene significance *P* value (p.GS.total), module membership value for each module (MM.module) and module membership *P* value for each module (p.MM.module).
